# Type II Cells in the Human Carotid Body Display P2X7 Receptor and Pannexin-1 Immunoreactivity

**DOI:** 10.3390/biom15111523

**Published:** 2025-10-29

**Authors:** Marcos Anache, Ramón Méndez, Olivia García-Suárez, Patricia Cuendias, Graciela Martínez-Barbero, Elda Alba, Teresa Cobo, Iván Suazo, José A. Vega, José Martín-Cruces, Yolanda García-Mesa

**Affiliations:** 1 Grupo SINPOS, Departamento de Morfología y Biología Celular, Universidad de Oviedo, 33003 Oviedo, Spain; uo298581@uniovi.es (M.A.); uo298626@uniovi.es (R.M.); garciaolivia@uniovi.es (O.G.-S.); cuendiaspatricia@uniovi.es (P.C.); gracielamartinezbarbero@gmail.com (G.M.-B.); ivan.suazo@uautonoma.cl (I.S.); javega@uniovi.es (J.A.V.); pepe3214@gmail.com (J.M.-C.); 2 Master ENDORE, Universidad de Santiago de Compostela, 15705 Santiago de Compostela, Spain; 3 Instituto de Investigación Sanitaria del Principado de Asturias, ISPA, 33011 Oviedo, Spain; 4 Instituto de Neurociencias Vithas, 28010 Madrid, Spain; eldamaselda@gmail.com; 5 Servicio de Neurología, Hospital Clínico San Carlos, 28040 Madrid, Spain; 6 Departamento de Cirugía y Especialidades Médico-Quirúrgicas, Universidad de Oviedo, 33003 Oviedo, Spain; teresacobo@uniovi.es; 7 Instituto Asturiano de Odontología, 33006 Oviedo, Spain; 8 Facultad de Ciencias de la Salud, Universidad Autónoma de Chile, Santiago 8330015, Chile

**Keywords:** carotid body, type II glomus cells, nerves fibre terminals, P2X7 purinergic receptor, pannexin 1 channel, immunohistochemistry, human

## Abstract

The carotid body is a peripheral chemoreceptor that consists of clusters of chemoreceptive type I cells, glia-like type II cells, afferent and efferent nerves, and sinusoidal capillaries and arterioles. Cells and nerves communicate through reciprocal chemical synapses and electrical coupling that form a “tripartite synapse,” which allows for the process of sensory stimuli within the carotid body involving neurotransmission, autocrine, and paracrine pathways. In this network there are a variety of neurotransmitters and neuromodulators including adenosine 5′-triphosphate (ATP). Carotid body cells and nerve fibre terminals express ATP receptors, i.e., purinergic receptors. Here we used double immunofluorescence associated with laser confocal microscopy to detect the ATP receptor P2X7 and pannexin 1 (an ATP permeable channel) in the human carotid body, as well as the petrosal and cervical sympathetic ganglia. Immunofluorescence for P2X7r and pannexin 1 forms a broad cellular network within the glomeruli of the carotid body, whose pattern corresponds to that of type II cells. Moreover, both P2X7r and pannexin 1 were also detected in nerve profiles. In the petrosal ganglion, the distribution of P2X7r was restricted to satellite glial cells, whereas in the cervical sympathetic ganglion, P2X7r was found in neurons and glial satellite cells. The role of this purinergic receptor in the carotid body, if any, remains to be elucidated, but it probably provides new evidence for gliotransmission.

## 1. Introduction

The carotid body (CB) is a principal peripheral chemoreceptor [1,2] located bilaterally at the bifurcation of the common carotid artery. Structurally, it consists of clusters of neuron-like chemoreceptive cells called type I cells in intimate association with glial-like cells called type II or supporting cells; clusters of type I cells and type II cells are arranged around the many sinusoidal capillaries and arterioles within the CB [3]. The CB is innervated by glossopharyngeal petrosal afferents, as well as by sympathetic efferents that originate in the sympathetic superior cervical ganglion [4,5]. Variations in blood levels of oxygen, carbon dioxide, pH, some metabolites and hormones, as well as inflammation mediators are detected by type I cells and convoyed to the central nervous system, where they play a key role in cardiorespiratory and metabolic regulation (see [2]).

The communication between cells and nerves within the CB is multidirectional. There is evidence for reciprocal chemical synapses and electrical coupling between type I cells and afferent nerve fibres as well as between neighbouring type I cells [6,7,8]. In addition, functional relationships exist between nerves and type II cells, and between type I and type II cells (see [9]). This complex network forms the so-called “tripartite synapse”, which refers to the relationships between type I cells, type II cells, and afferent nerve terminals [10]. The tripartite synapse allows for the processing of sensory stimuli within the CB involving both autocrine and paracrine pathways [11].

Type I cells are the sensors and transducers of chemical stimuli, and when they depolarize in response to appropriate stimuli [1], they respond releasing a variety of both excitatory and inhibitory neurotransmitters and neuromodulators [12,13,14]. These molecules mainly present in nerve afferent terminals through the counterpart receptors, but also in type I and type II cells. Among these neurotransmitters is adenosine 5′-triphosphate (ATP).

The extra-mitochondrial ATP released by type I cells of the CB can function as an excitatory [14,15] or inhibitory [16,17] neurotransmitter. ATP binds on P2X2 and P2X3 purinergic receptors present in the afferent terminals [18,19,20,21] and in the majority (>65%) of neurons in the sensory ganglia, including the petrosal ganglion [18,22,23,24]. However, ATP can also act on the P2Y1 and P2Y2 purinergic receptors localized in type II cells [15,25,26,27], leading to the further release of ATP opening of large-pore ATP-permeable pannexin-1 channels [15,27,28]. Both P2Y channels and pannexin 1 are expressed in type II cells [29,30,31]. Altogether, these data suggest that type II cells may participate in the neurochemical networks of the CB via ‘gliotransmission,’ especially throughout pathways involving ATP [31,32,33].

As far as we know, the only P2X receptors detected in nerves supplying the mammalian CB are P2X2 and P2X3, whereas the sensory ganglia of the glossopharyngeal nerve expressed at least four different subtypes: P2X2, P2X3, P2X4, and P2X7 [34]. Interestingly, while P2X2-P2X3 were detected in the neurons, P2X7R was restricted to the satellite glial cells [22,35,36,37], together with pannexin 1 [38]. P2X7 receptor and pannexin 1 form a dynamic channel pore responsible for calcium influx in the cells [39,40].

Type II cells of the CB and satellite glial cells in sensory ganglia are closely related, since both originate from the neural crest and express some common antigens, such as glial fibrillary acidic protein, S100 protein, vimentin, or nestin [41,42,43]. Here we hypothesized that the glia-like type II cells in the CB express the P2X7 receptor and pannexin 1 like the satellite glial cells in the sensory ganglia. The study would provide new data on the putative role of type II cells in the purinergic neurotransmission, as well as in ATP-mediated paracrine communication within the CB.

## 2. Materials and Methods

### 2.1. Human Tissue Samples

Tissue samples containing the common carotid bifurcation and the CB were obtained during organ removal for transplantation from eight donors (five males, three females; 38–68 years) who died in traffic accidents at the Hospital Universitario Central de Asturias (Oviedo, Spain). Petrosal ganglia (*n* = 4) and superior cervical sympathetic ganglia (*n* = 4) were also dissected and included in the study. Samples were rinsed in saline at 4 °C, fixed in 10% formaldehyde in 0.1 M phosphate-buffered saline (PBS, pH 7.4) for 24 h at 4 °C, dehydrated, and paraffin-embedded using standard protocols. The blocks were cut 10 μm thick and mounted on gelatine-coated slides.

All procedures were conducted in agreement with Spanish law and the principles of the Helsinki Declaration II. The sections are preserved at the Department of Morphology and Cell Biology, University of Oviedo Biobank (National Registry of Biobanks, Ref. C-0001627), authorized by the Spanish Ministry of Economy and Competitiveness (30 November 2012) and can be used exclusively for research purposes.

### 2.2. Fluorescence and Double Immunofluorescence

For simple immunofluorescence, deparaffinized and rehydrated sections were incubated with anti-P2X7r or anti-pannexin 1 antibody. The antibodies against P2X7r and pannexin 1 recognize specific epitopes within these proteins (supplier notice). Rehydration was carried out with Tris-HCl buffer (0.05 M, pH 7.5) containing 0.1% bovine serum albumin (BSA; Sigma-Aldrich, St. Louis, MO, USA) and 0.1% Triton X-100 (Sigma-Aldrich). The non-specific binding was blocked with 10% fetal calf serum (F2442, Sigma-Aldrich) in Tris-buffered saline (TBS) for 30 min. Sections were incubated overnight in a dark humid chamber with primary antibodies (Table 1), and after rinsing in TBS were incubated sequentially with CFL488-conjugated bovine anti-rabbit IgG (1:200; sc-362260, Santa Cruz Biotechnology, Heidelberg, Germany) for 1 h. Nuclear counterstaining was performed using 4′,6-diamidino-2-phenylindole (DAPI; 10 ng/mL) and the sections mounted in Fluoromount-G mounting medium (Southern Biotech, Birmingham, AL, USA).

Four double immunofluorescence sections were incubated overnight at 4 °C in a dark, humid chamber with a 1:1 (*v*/*v*) mixture of polyclonal antibodies anti-P2X7r, or anti-pannexin 1 and monoclonal antibodies against neurofilament protein (NFP), synaptophysin (SYN), or S100 protein (S100P) diluted in Tris-HCl buffer (Dako, Glostrup, Denmark), supplemented with 0.1% BSA, 0.2% fetal calf serum, and 0.1% Triton X-100 (Table 1). After rinsing in TBS, sections were incubated sequentially with CFL488-conjugated bovine anti-rabbit IgG (Santa Cruz Biotechnology) for 1 h, followed by Cy™3-conjugated donkey anti-mouse IgG (1:100; Jackson ImmunoResearch, Baltimore, MD, USA) for 1 h. Both steps were performed in a dark, humid chamber at room temperature, with phosphate-buffered saline containing Tween 20 washes between incubations. Nuclear counterstaining was performed using DAPI (10 ng/mL) and the sections were mounted in Fluoromount-G mounting medium (Southern Biotech, Birmingham, AL, USA).

Negative controls were processed in parallel by omitting the primary antibodies, substituting them with non-immune rabbit or mouse sera. Moreover, sections were incubated with dilutions of the primary antibodies for P2X7r and pannexin 1 specifically preabsorbed (1 µg/mL). Under these conditions, no specific immunoreactivity was detected (Supplementary Material Figure S1).

Confocal imaging was performed using a Leica TCS SP8 X confocal microscope coupled to a Leica DMI8 fluorescence microscope. Our images have been acquired using a 20×/NA 0.75 oil immersion objective, a 40×/NA 1.30 oil immersion objective, and a 488 nm and 545 nm laser excitation wavelength. Under these parameters, the optical section resolution of our images has been 1 μm for the 20× objective and 800 nm for the 40× objective. Images were acquired with Leica Application Suite X software (version 1.8.1; Leica Microsystems CMS GmbH, Wetzlar, Germany) at the Optical Microscopy and Image Processing Unit, University of Oviedo, and analysed with ImageJ software (version 1.43g; McMaster Biophotonics Facility, Hamilton, ON, Canada).

### 2.3. Quantitative Analysis

Quantitative image analysis was performed on the CB processed for all the antigens investigated using an automated system (Quantimet 550, Leica Microsystems; QWIN software v3). Whole sections were scanned at 50× magnification using a SCN400F scanner (Leica Biosystems™, PubCompare, Sion, Switzerland); the images were processed with the SlidePath Gateway LAN program (Leica Biosystems™) at the Histopathology Laboratory, University Institute of Oncology of the Principality of Asturias. Ten randomly selected fields (5 mm^2^ each) were analysed per section in five sections spaced 100 μm apart, totalling 400 fields. The immunoreactive area for S100P was defined as 100% of the type II cell area. The merge areas of S100P + SYN, S100P + P2X7r, and S100P + pannexin 1 were quantified to estimate the percentage of type II cells that express P2X7r or pannexin 1. The merge areas of SYN + P2X7r and SYN + pannexin 1 were also quantified. The results correspond to the immunofluorescent area per mm^2^ and are presented as mean ± SEM.

In parallel, nerve fibre terminal density was quantified from NFP-immunofluorescence in the same sampling scheme (10 fields/section, five sections, 400 fields). The area of NFP immunoreactivity was considered to be 100% of the nerve fibre terminal profiles, and the overlap of NFP with P2X7r or pannexin 1 was taken as the proportion of nerve fibre terminals co-expressing the respective markers. The results correspond to immunofluorescent area per mm^2^.

Quantitation was performed in duplicate by two independent observers, and the results were homogeneous between replicates.

## 3. Results

### 3.1. Immunolocalization of P2X7r and Pannexin 1 in the Human Carotid Body

**Figure 1:**
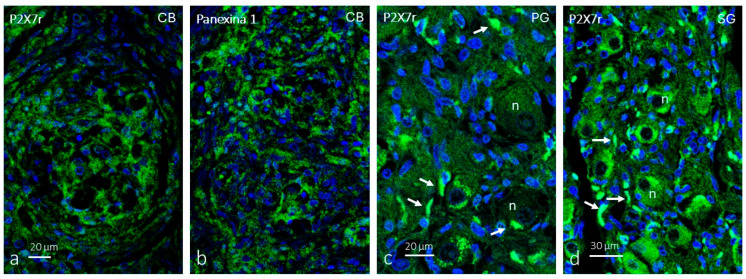
Immunofluorescence-based detection of P2X7r in the human carotid body (CB), petrosal ganglia (PG) and cervical sympathetic ganglia (SG). Within the CB, P2X7r was detected in cells whose morphology and arrangement suggest they are type II cells (**a**,**b**). In the PG (**c**) and SG (**d**), intense immunofluorescence for P2X7r was observed in the satellite glial cells (white arrows) and in a subpopulation of SG neurons; in the PG, weak immunofluorescence for P2X7r was detected in some neuronal bodies. n: neuron bodies. Scale bar is the same for (**a**,**b**). Objective: 20×/0.75 oil; pinhole: 1; XY resolution: 156 nm; and Z resolution: 334 nm.

Immunofluorescence for P2X7r (Figure 1a) and pannexin 1 (Figure 1b) formed a broad cellular network whose morphological pattern corresponded to that of the arrangement of type II cells within the CB glomeruli. In the petrosal ganglion, strong immunofluorescence for P2X7r was detected in the labelling of satellite glial cells, whereas the neurons were not reactive, although a small subpopulation (around 5–10%) displayed a weak but specific immunofluorescence. In the cervical sympathetic ganglion, P2X7r immunofluorescence was found in the satellite glial cells and in a subpopulation of neurons of about 30–35% (Figure 1d). In both the CB and ganglia, the immunofluorescence pattern was cytoplasmic and not in the cell membrane, as it would be expected due to the cellular localization of P2X7r.

**Figure 2:**
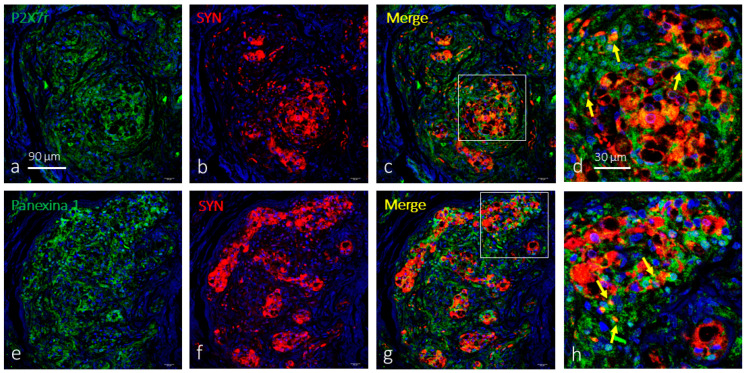
Immunofluorescence-based detection of P2X7r (**a**–**d**) and pannexin 1 (**e**–**h**) in the human carotid body. Cells displaying P2X7r immunofluorescence (green fluorescence in (**a**)) and pannexin 1 immunofluorescence (green fluorescence in (**e**)) form a dense meshwork throughout glomic glomeruli consistent with their presence in type II cells. Type I cells showed strong immunofluorescence for SYN (red fluorescence in (**b**,**f**)). Merger (yellow fluorescence) of P2X7r (**c**,**d**) or pannexin 1 (**g**,**h**) with SYN, showing cell segregation. The occasional merger of P2X7r and pannexin 1 and SYN was found in disperse drops (yellow arrows). Scale bar is the same for (**a**–**c**,**e**–**g**); scale bar is the same for (**d**,**h**). Objective: 20×/0.75 oil; pinhole: 1; XY resolution: 156 nm; and Z resolution: 334 nm.

To exclude that P2X7r and pannexin 1 are present in type I cells of the CB, a double immunolabeling of these proteins with others specific to type I cells (synaptophysin), type II cells (S100 protein), and nerve fibre terminals (neurofilament proteins) was performed. The simultaneous detection of P2X7r with synaptophysin showed that both are located in segregated cell populations within the CB, so it can be assured that P2X7r was not localized in type I cells. P2X7r-positive cells (type II cells) are arranged around the synaptophysin-positive cells (type I). However, at the interface of type I and type II cells and in some isolated “drops profiles,” co-localization of both proteins appears (Figure 2a–d). The results for the co-detection of pannexin 1 and synaptophysin were identical (Figure 2e–h).

**Figure 3:**
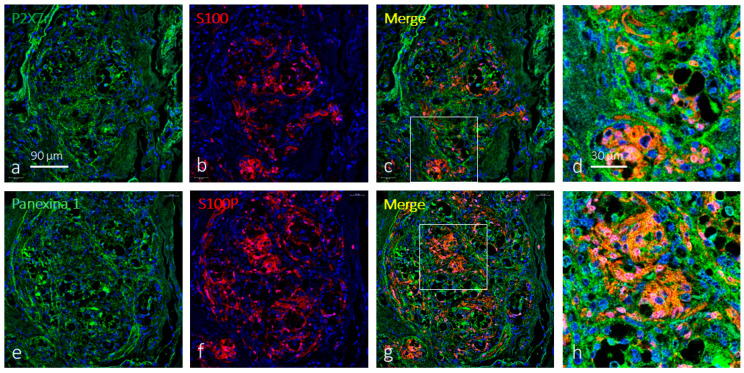
Immunofluorescence-based detection of P2X7r (**a**–**d**) and pannexin 1 (**e**–**h**) in the human carotid body. Cells displaying P2X7r immunofluorescence (green fluorescence in (**a**)) and pannexin 1 immunofluorescence (green fluorescence in (**e**)) as well as S100 protein immunofluorescence (S100P; red fluorescence in (**b**,**f**)) form a dense meshwork throughout glomic glomeruli consistent with their presence in type II cells. The merger (yellow fluorescence) of P2X7r (**c**,**d**) or pannexin 1 (**g**,**h**) with S100P show cell colocalization of P2X7r and pannexin 1 with S100P, thus confirming that both are localized in type II cells. Scale bar is the same for (**a**–**c**,**e**–**g**); scale bar is the same for (**d**,**h**). Objective: 20×/0.75 oil; oil; pinhole: 1; XY resolution: 156 nm; and Z resolution: 334 nm.

Then, to confirm that P2X7r and pannexin 1 are in type II cells, a simultaneous co-localization study with S100 protein was carried out. S100 protein immunofluorescent cells form a network whose morphology is consistent with the labelling of type II cells, and is co-localized with P2X7r (Figure 3a–d) and pannexin 1 (Figure 3e–h). In addition, there are cells within the glomeruli of the CB that are positive for P2X7r and pannexin 1 that do not express the S100 protein; that is, based only on immunofluorescence, P2X7r and pannexin 1 are present in cells of the CB other than type II cells.

**Figure 4:**
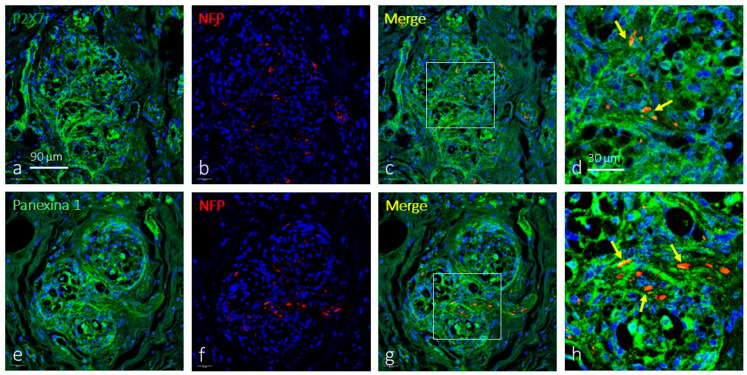
Immunofluorescence-based detection of P2X7r (**a**–**d**) and pannexin 1 (**e**–**h**) in the human carotid body. P2X7r (green fluorescence in (**a**)) and pannexin 1 (green fluorescence in (**e**)) within the CB are present in a cell meshwork consistent with its presence in type II cells. Nerve fibre terminal profiles scattered through the CB display immunofluorescence for neurofilament protein (NFP; red fluorescence in (**b**,**f**)). Merge (yellow fluorescence) of P2X7r (**c**,**d**) and pannexin 1 (**g**,**h**) with NFP show the presence of both proteins in some nerve fibre terminal profiles (yellow arrows). Scale bar is the same for (**a**–**c**,**e**–**g**); scale bar is the same for (**d**,**h**). Objective: 20×/0.75 oil; oil; pinhole: 1; XY resolution: 156 nm; and Z resolution: 334 nm.

We also investigated whether P2X7r and pannexin 1 are found in the nerve fibre terminals that innervate the CB. The co-localization of P2X7r and pannexin 1 with neurofilament proteins shows that all neurofilament-positive nerve fibre terminals express both proteins, especially in nerve fibres located in the most peripheral part of the CB (Figure 4).

**Scheme 1:**
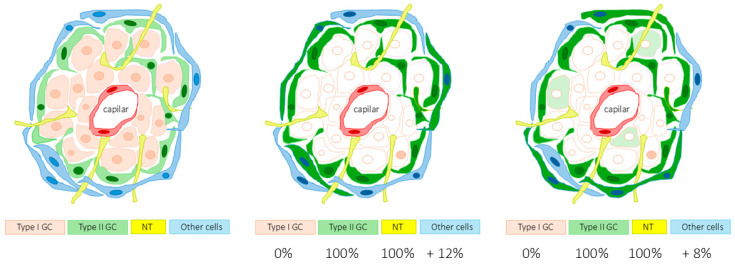
Percentage of area occupied by P2X7r and pannexin 1 in the carotid body in type II glomus cells (green) and nerve fibre terminals (yellow). Immunofluorescence was also detected in cells other than type I and type II cells (see also Section 2 and Figure S2). Type I glomus cells lacked both P2X7r and pannexin 1. This diagram completes Table 2 and shows the distribution of immunofluorescence for P2X7r and pannexin 1 in a relatively manner.

The area occupied by the specific immunoreaction of P2X7r was 48.6 ± 1.3% of the total section, and no area occupied by the merger of SYN + P2X7r was observed, although a residual merger was observed; the merger of NFP + P2X7r was 100%, and 12.3 ± 1.8% of the total section was P2X7r positive and SYN, NFP and S100 negative, thus indicating that P2X7r is present in a cells other than type I, type II, or nerve fibre terminals (Table 2 and Scheme 1). The quantitative results for pannexin 1 in the CB paralleled those of P2x7r (Table 2 and Scheme 1).

Therefore, P2X7r and pannexin 1 are absent from type I glomus cells of the human CB while they are present in type II cells and nerve fibres. Values were similar in all the samples analysed, without gender- or age-dependent variations.

### 3.2. Immunolocalization of P2X7r and Pannexi-1 in Petrosal and Cervical Sympathetic Ganglia

**Figure 5:**
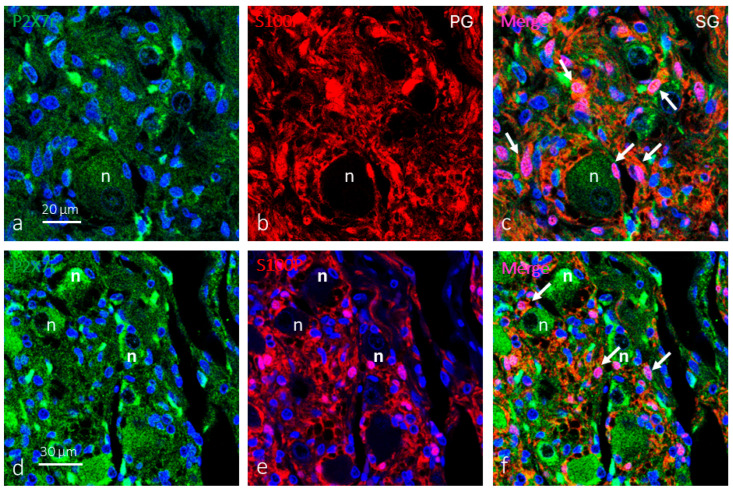
Immunofluorescence-based detection of P2X7r in the petrosal (**a**–**c**) and cervical sympathetic ganglia (**d**–**f**). In both petrosal and cervical sympathetic ganglia P2X7r, immunofluorescence (green fluorescence in (**a**) and (**d**), respectively) was detected in satellite glial cells (S100 protein positive; red fluorescence in (**b**,**e**)). The merger (purple fluorescence; white arrows) of P2X7r and S100P shows co-localization of both proteins. P2X7r was also detected in a subpopulation of sensory and sympathetic neurons. n: neurons. Scale bar are identical for (**a**–**c**) and for (**d**–**f**). Objective: 40×/1.30 oil; pinhole: 1; XY resolution: 156 nm; and Z resolution: 334 nm.

In the petrosal ganglion of the glossopharyngeal nerve, the soma of the neurons that originate the afferent nerve fibres innervating the CB were devoid of immunofluorescence for P2X7r although about 5% displayed a weak granular immunofluorescence. By contrast, P2X7r immunofluorescence was detected in most glial satellite cells (Figure 5a–c). In the cervical sympathetic ganglion, a larger neuronal subpopulation (about 30–35%) was P2X7r positive (Figure 5d–f), and the satellite glial cells displayed strong P2X7r immunofluorescence. The identification of the neurons in the petrosal ganglia and upper cervical sympathetic ganglia was performed using only morphological criteria.

Therefore, based on the present results of immunofluorescence in the human CB, P2X7r and pannexin 1 are absent from type I glomus cells and restricted to type II glomus cells.

## 4. Discussion

**Figure 6:**
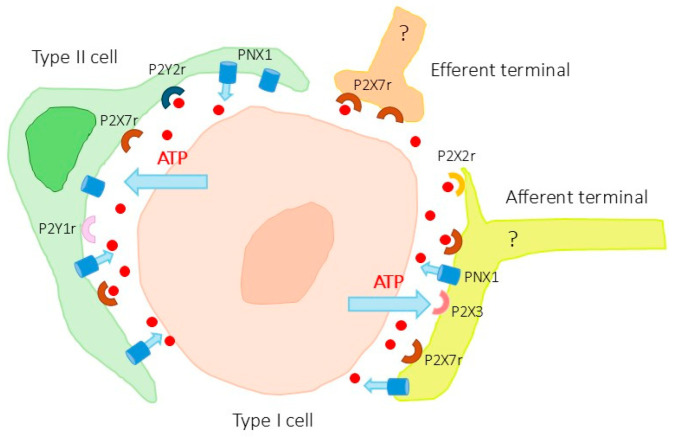
Schematic representation of ATP-mediated neurotransmission–gliotransmission. Type I cells release ATP that acts on P2X2 and P2X3 receptors on afferent terminals, and on P2Y1, P2Y2, and P2X7 receptors on type II cells. In turn, the pannexin 1 (PNX1) channels present in type II cells would release more ATP from these cells. The presence of P2X7 and pannexin 1 in the afferent and efferent terminals should be taken with caution pending physiological and pharmacological studies.

The CB is organized into glomeruli, which are clusters of chemoreceptor cells (type I cells) and supporting glia-like cells (type II cells) in close contact with afferent nerve endings and a profuse network of capillaries [3]. Type I cells release several classical neurotransmitters, but also ATP [12,13,14,21,44]. It is generally accepted that ATP, released from type I cells during chemotransduction, is the principle excitatory neurotransmitter that initiates the chemoreflex by acting on purinergic receptors on the afferent nerve terminals [2,10,11,12,44]. It is now known that ATP released by type I cells acts on purinergic receptors [45] located in afferent nerve endings [18,19,20,21,22,23,24] and in type II cells [25,26,27,28]. This system appears to be a potential paracrine mechanism for ATP signalling at the chemosensory synapse, and fits within the concepts of tripartite synapses and gliotransmission [10,11,33] (Figure 6). In addition, type II cells express pannexin 1 membrane channels related to ATP release [11,29,30,31] and which are a part of the ATP-induced ATP release hypothesis [11].

The present research was designed to analyse whether the human CB expresses ATP channels of type pannexin 1 and ATP receptors of type P2X7. The study was conducted on human material using immunofluorescence techniques associated with confocal laser microscopy. For the identification of type I and type II cells of the CB, as well as afferent and efferent nerve fibre terminals, antibodies that had already demonstrated their specificity in this organ and that function in human material fixed and included in paraffin were used. In all the samples analysed, with no apparent differences in relation to age or sex, specific immunofluorescence was detected with a fine granular cytoplasmic pattern and not a membrane, as would be expected due to the location of both proteins in the cell membrane. The thickness of the sections on which the study was carried out is probably responsible for this pattern. To demonstrate the presence of P2X7r and pannexin 1 in membranes, immunocytochemistry work with electron microscopy is necessary.

In our study we detected P2X7R and pannexin 1, two molecules linked to ATP neurotransmission, in type II glomic cells, but not in type I chemosensory cells. The presence of ATP-permeable pannexin 1 channels in type II cells in the CB of mammals had already been demonstrated previously [11,29,30,31]. Our findings confirm that pannexin 1 is also expressed in type II cells of the human CB. In contrast, here we demonstrate for the first time the presence of P2X7 receptors in type II cells of the human CB. As far as we know, this receptor has not been described in this location, although its presence in the glial satellite cells of the sensory ganglia, which are related to CB type II cells, has been demonstrated. It is important to note that this purinergic receptor is expressed in the glial satellite cells of the sensory ganglia and not in the neurons [32,35,36,37].

Type II cells have historically been considered supporting cells; however, many now accept that they act to modulate CB chemotransmission through paracrine mechanisms [11,31,46] especially those involving ATP. Moreover, type II cells, in absence of type I cells, can trigger afferent firing via ATP release [47]. Activation of purinergic channels in type II cells leads to the opening of a large-pore pannexin 1 channel and further releases ATP [27,28,31]. Therefore, Zang et al. [28] proposed that type II cells of the CB function as ATP amplifiers during chemotransduction via paracrine activation of P2Y receptors and pannexin 1 channels, leading to ATP-induced ATP release. Whether P2X7r functions similarly to P2Y channels in type II cells will need to be clarified in future studies. The function of P2X7r in type II cells of the human CB, if any, is still unknown. In the spinal ganglia, they modulate different intracellular pathways, including pro-inflammatory and tumour-promoting cascades (see [48]). Importantly, pannexin 1 channels, in addition to mediating actions linked to ATP, mediate other actions of other neurotransmitters with 5-HT [29].

Apart from type II cells in the CB, we have observed immunofluorescence for P2X7r in nerve fibre terminals, and therefore we investigate whether they are also detected in the neurons of the petrosal and cervical sympathetic ganglia, which is where the somas of the neurons that innervate the CB are located. P2X7r was located in the satellite glial cells of the sensory ganglia, as earlier reported [22,34,35,36,37], but only at residual levels in a small subpopulation in sensory neurons. Conversely, in the cervical sympathetic ganglia, P2X7r was predominantly neuronal, as previously observed [49,50]. Therefore, the efferent and afferent innervation of CB would contain P2X7r. Further studies are needed to definitively establish this issue; studies are in progress in our laboratory to co-localize P2x7r and tyrosine hydroxylase to demonstrate whether or not postganglionic sympathetic neurons express P2X7r. On the other hand, although in our work we have not analysed the localization of pannexin 1 in sensory ganglia, previous studies have located it in satellite glial cells [38,51,52]. Regarding afferent and efferent nerve fibres, although we consider that P2X7r and pannexin 1 are in the axon, their presence in Schwann cells cannot be ruled out.

An interesting fact that should be highlighted is that pannexin 1 can also be activated by mechanical stretch [53]. Recently, we have observed that type II cells in the human CB express PIEZO1 and PIEZO2, two proteins that are part of mechanosensitive and mechanotransducer channels [54]. The connection between pannexin 1 and Piezo channels should be studied.

Altogether, the present results add new information to the reality that within the CB chemosensory complex, the glia-like type II cell might participate in a tripartite synapse, where purinergic neurotransmission is modulated. In turn ATP helps to coordinate activity within this network and thereby modulate afferent firing frequency during chemotransduction [33].

## 5. Conclusions

Type II cells of the human CB express P2X7r and pannexin 1 that are probably paracrine involved in the tripartite synapse, where neurotransmission is modulated. The functional implications of these findings need to be resolved in future studies.

## Figures and Tables

**Table 1:** Primary antibodies used.

Antigen	Origin	Dilution	Supplier
P2X7R	Rabbit	1:400	Alomone Labs ^1^
Pannexin 1	Rabbit	1:100	Alomone Labs ^1^
NFP (clone 2F11)	Mouse	1:200	Roche ^2^
Synaptophysin (clone 27G12)	Mouse	Prediluted	Leica Biosystems ^3^
S100P	Mouse	1:500	Dako ^4^

^1^ Jerusalem, Israel (anti-P2X7r: Cat# APR-004; control peptide P2X7r: Cat# PLP-PR004; anti-pannexin 1: Cat# ACC-234; control peptide pannexin 1: Cat# BLP-CC234); ^2^ Barcelona, Spain; ^3^ Madrid, Spain; ^4^ Glostrup, Denmark).

**Table 2:** Percentage of area occupied by P2X7r and pannexin 1 in the carotid body in type I glomus cells (merging SYN + P2X7r or pannexin 1), type II glomus cells (merging S100P + P2X7r or pannexin 1), and PIEZOs) and nerve fibre terminals (merging of NFP + P2X7r or pannexin 1).

P2X7r	Total: 48.6 ± 1.3% (100%)
	Type I glomus cells: 0.0–0.3%
	Type II glomus cells: 100%
	Nerve fibre terminals: 2.2 ± 0.3%: 100%
	Other cells: 12%
Pannexin 1	Total: 58.1 ± 5.7% (100%)
	Type I glomus cells: 0.0–0.1%
	Type II glomus cells: 100%
	Nerve fibre terminals: 3.0 ± 0.7%: 100%
	Other cells: 8%

## Data Availability

The original contributions presented in this study are included in the article/Supplementary Material. Further inquiries can be directed to the corresponding author.

## Supplementary Materials

The following supporting information can be downloaded at: https://www.mdpi.com/article/10.3390/biom15111523/s1, Figure S1: Control of the specificity of the immunoreactivity; Figure S2: The structural unit of the carotid body is the glomoid or glomerulus (delimited in the image on the left by the dotted lines), formed by groups of type I cells, also called glomic cells or chemoreceptor cells (red fluorescence), surrounded by type II cells, glial or sustentacular cells (green fluorescence).
