# Supplementary material for: Type II Cells in the Human Carotid Body Display P2X7 Receptor and Pannexin-1 Immunoreactivity
Source: Biomolecules. 2025 Oct 29;15(11):1523. doi: 10.3390/biom15111523 (PMC12649879; doi:10.3390/biom15111523)
Supplement: Supplementary file 1 [file biomolecules-15-01523-s001.zip › biomolecules-3909581-supplementary.pdf]

Supplementary Material

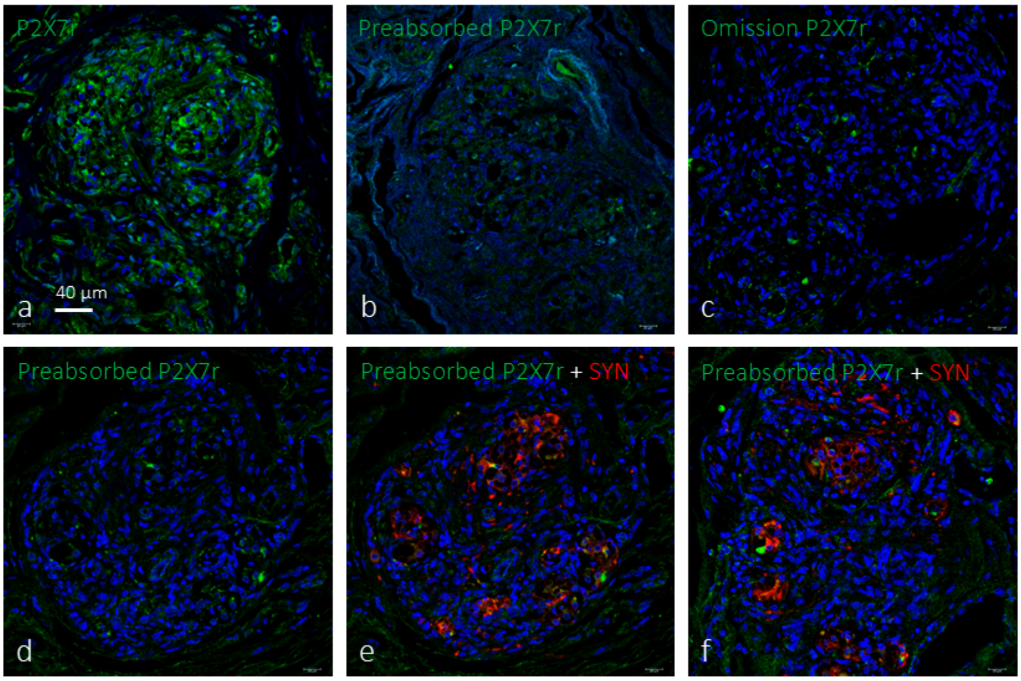

Figure S1. Control of the specificity of the immunoreactivity. The specificity of the immunoreactivity developed with the anti-P2X7r antibody used were carried out omitting the the incubation with the primary primary antibody, substituting it with non-immune rabbit or mouse sera, and incubating the sections with the diluted antibody (1:400) preabsorbed with the specific peptide (1 µg/mL). In the sections incubated with the anti-P2X7r antibody immunofluorescence was detected in typo II cells(a), whereas the preadsorption (b,d) or omission of the anti-P2X7 antibody (c) resulted in a total abolition of immunoreactivity (only residual labeling persists). The immunoreactivity was preserved for the other antigens investigated (e,f).

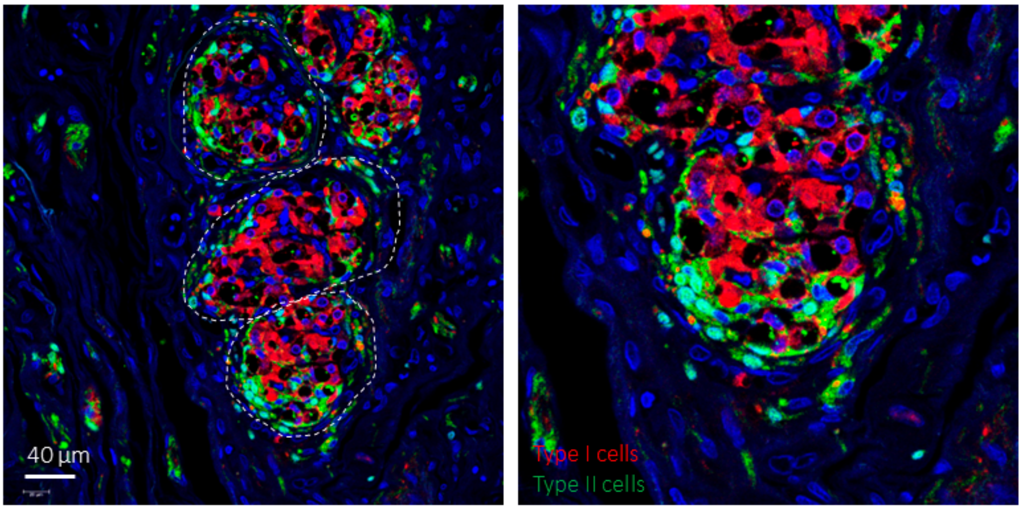

Figure S2. The structural unit of the carotid body is the glomoid or glomerulus (delimited in the image on the left by the dotted lines), formed by groups of type I cells, also called glomic cells or chemoreceptor cells (red fluorescence), surrounded by type II cells, glial or sustentacular cells (green fluorescence). The grouping of glomeruli forms the lobules. In addition to these two main

cell types, there are also endothelial cells in capillaries, and in the connective tissue septa that separate the glomeruli and lobes from each other are fibroblasts, histiocytes, and macrophages.
